# Volar Plate Avulsion Injury

**Published:** 2016-06-01

**Authors:** Ashish Pattni, Matt Jones, Sameer Gujral

**Affiliations:** Department of Plastic Surgery, Royal Devon & Exeter Hospital, Exeter, Devon, England

**Keywords:** volar plate, avulsion, fracture, dislocation, splint

## DESCRIPTION

A right-hand-dominant male sustained a closed hyperextension finger injury catching a basketball, presenting with pain, swelling, and bruising over the volar proximal interphalangeal joint (PIPJ) of the finger. Radiograph demonstrated an avulsion fracture at the base of the proximal middle phalanx and was managed in an extension-blocking splint.

## QUESTIONS

1. **What is the anatomy of the volar plate?**
2. **What is a volar plate injury?**
3. **How are volar plate injuries assessed and classified?**
4. **What is prognosis following volar plate injury?**

## DISCUSSION

**Figure 1:**
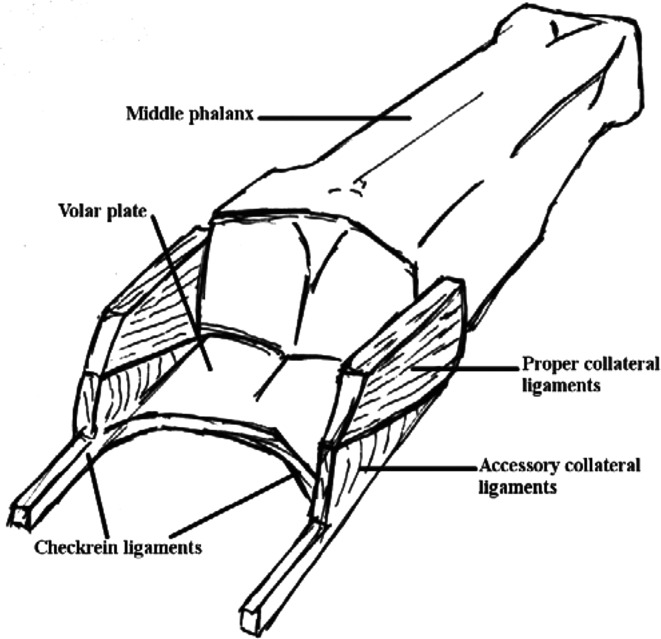
Anatomy of the volar plate.

The PIPJ is a synovial hinge joint, allowing flexion and extension (range = 0° to 100°-110°). The volar plate helps maintain stability of the PIPJ in the anteroposterior plane and prevents PIPJ hyperextension. The volar plate is a multilayered condensation of fibrocartilagenous tissue lying between the flexor tendons and the palmar PIPJ capsule. It originates from the proximal phalanx and inserts onto the middle phalanx. At its proximal origin, it has extensions called “checkrein ligaments,” which attach to the periosteum of the proximal phalanx adjacent to the distal end of the A2 pulley (Fig 1). The collateral ligaments (ulnar and radial) also contribute to the stability of the PIPJ.^1^

**Figure 2:**
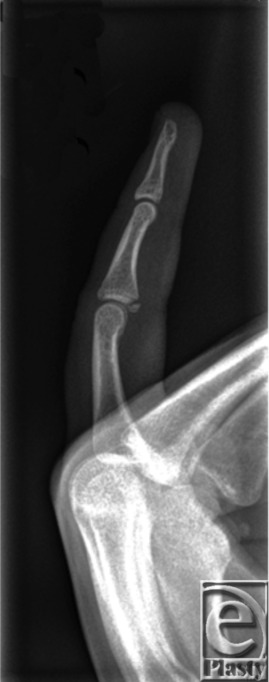
Radiograph demonstrating a volar plate avulsion fracture (Eaton type 3a).

Forced, sudden hyperextension and occasionally crush injuries of the PIPJ can result in partial or complete volar plate and collateral rupture. Volar plate injury occurs more commonly in younger patients, particularly those involved in hand/contact sports. In most instances, volar plate rupture occurs distally, at the weaker fusion with the middle phalanx, whereas the proximal stronger checkrein ligaments rarely rupture. Volar plate injury can occur with an avulsion fracture, most commonly at the volar base of the middle phalanx (Fig 2). Subluxation/dislocation of the PIPJ may also occur.^2^

**Figure 3:**
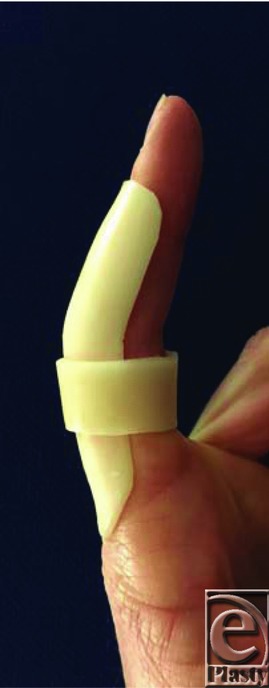
Immobilization of a volar plate injury with an extension-blocking splint (proximal interphalangeal joint in 20°-30° flexion).

Diagnosis of volar plate injury is based on history and examination. There is tenderness maximally over the volar PIPJ, pain on passive hyperextension of the injured finger, possible PIPJ instability, and loss of pinch power. Radiograph can diagnose avulsion fracture at the base of the volar middle phalanx and identify PIPJ subluxation/dislocation. There are several classification systems; two of the most useful are (i) Eaton's and (ii) the Keifhaber-Stern classifications (Table 1).^3^^,^^4^ For Eaton classification type 1-3a and Keifhaber-Stern classification *stable* or *tenuous*, conservative management is most appropriate. This implies that injuries involve less than 30% to 40% PIPJ surface and are reducible in less than 30° flexion. In these instances, splinting with extension-blocking immobilization (PIPJ in 20° to 30° flexion) is commenced (Fig 3). Mobilization is initiated with a protective splinting as swelling subsides.^3^^,^^4^ For Eaton type 3b and Keifhaber-Stern *unstable*, surgical treatment is recommended. This implies that injuries involve more than 40% to 50% PIPJ surface and are irreducible or require more than 30° flexion to reduce the fragment. In these and in open injuries, open reduction and internal fixation using various techniques are required.^3^^,^^4^ This can include volar plate arthroplasty, screw fixation of the volar fragment, and/or k-wire to temporarily stabilize the PIPJ. On occasion, comminuted intra-articular fractures may be managed by dynamic external fixation traction, for example, a Suzuki frame.^5^

Conservative management for closed, stable volar plate injury (eg, Eaton type 1-3a, or Keifhaber-Stern *stable or tenuous*) with splinting and hand therapy appears to lead to good outcomes.^6^ For those requiring operative intervention, range-of-motion outcomes appear better if surgery is performed within 4 weeks of injury (average 85° PIPJ vs average 61° if operated on after 4 weeks of injury).^7^ Injuries left untreated may result in chronic PIPJ dysfunction causing subluxation at the joint, rendering the patient with a swan neck deformity. Consequences can also include flexion contracture with reduced range of movement and weakness of grip.^8^ These may ultimately then need arthrodesis or arthroplasty.

Volar plate injury encompasses a spectrum of soft tissue and bony injury at the PIPJ commonly due to hyperextension. Understanding of the anatomy of the volar plate and use of classification systems can guide appropriate management. Mismanagement or delayed treatment can result in chronic PIPJ problems to include stiffness, subluxation, and swan neck deformities, leading to weakness of grip and poor function.

## Figures and Tables

**Table 1:** Classification systems of volar plate injuries

	Eaton classification of volar plate injuries^3^
Type 1	Avulsion of the volar plate without a fracture or dislocation
Type 2	Complete dorsal dislocation without fracture and avulsion of the volar plate
Type 3a	Fracture-dislocation with <40% PIPJ surface with dorsal portion of the collateral ligaments remaining attached to the middle phalanx (stable)
Type 3b	Fracture-dislocation with >40% PIPJ surface with little or no ligament remaining attached to the middle phalanx (unstable)
	Keifhaber-Stern classification of volar plate injuries^4^ (modification of Hastings classification)
Stable	Avulsion fracture involving <30% articular base of the middle phalanx
Tenuous	Avulsion fracture involving 30%-50% articular base of the middle phalanx; reduces with <30° of flexion
Unstable	Avulsion fracture involving <50% articular base of the middle phalanx but requires >30° flexion to maintain reduction

PIPJ indicates proximal interphalangeal joint.
